# Complex evaluation of coagulation, fibrinolysis, and inflammatory cytokines in SARS-CoV-2 infected pregnant women: a prospective, case-control study

**DOI:** 10.3389/fimmu.2025.1556878

**Published:** 2025-04-15

**Authors:** Zsuzsa Bagoly, Eszter Lilla Tóth, Rita Orbán-Kálmándi, Linda Lóczi, Tamás Deli, Olga Török, Bence Kozma, Sándor Baráth, Parvind Singh, Zsuzsanna Hevessy, Judit Tóth, Éva Katona, Szabolcs Molnár, Zoárd Tibor Krasznai

**Affiliations:** ^1^ Division of Clinical Laboratory Sciences, Department of Laboratory Medicine, Faculty of Medicine, University of Debrecen, Debrecen, Hungary; ^2^ Department of Obstetrics and Gynecology, Faculty of Medicine, University of Debrecen, Debrecen, Hungary; ^3^ Doctoral School of Molecular Medicine, University of Debrecen, Debrecen, Hungary; ^4^ Healthcare Industry Institute, Faculty of Pharmacy, University of Debrecen, Debrecen, Hungary; ^5^ Kálmán Laki Doctoral School, University of Debrecen, Debrecen, Hungary; ^6^ Department of Laboratory Medicine, Faculty of Medicine, University of Debrecen, Debrecen, Hungary

**Keywords:** COVID-19, hemostasis, fibrinolysis, pregnancy, postpartum hemorrhage

## Abstract

**Background:**

Given the physiological hemostasis changes during pregnancy, limited data exists on COVID-19-induced inflammatory response and hemostasis alterations in pregnant women.

**Objectives:**

To test a comprehensive set of hemostasis and inflammatory cytokines in pregnancies with/without COVID-19 and correlate results with maternal and perinatal outcomes.

**Patients/methods:**

In this observational case-control study, 100 women with acute COVID-19 at 24-40 gestational weeks (COVID-19+ group), and 100 healthy, age- and gestational week-matched, SARS-CoV-2 negative pregnant women (32 with proven recovery of COVID-19) were enrolled. All women were outpatients with mild/no symptoms at admission. Detailed hemostasis (fibrinogen, FVIII, FXIII, VWF, plasminogen, α2-plasmin inhibitor, PAI-1, thrombin generation, clot lysis, D-dimer) and inflammatory cytokine/chemokine panels were performed. Clinical parameters of pregnancy, labor and postpartum period were registered.

**Results:**

COVID-19+ women exhibited significantly lower FVIII, FXIII, plasminogen, higher VWF levels, decreased peak thrombin and enhanced clot lysis vs. controls. Despite mild/no symptoms, significantly elevated cytokine levels, including IL-6, INF-γ, MCP-1, and IL-18 were observed in COVID-19+ pregnancies, associated with distinct hemostasis alterations. Admission IL-1β, and IL-33 were significantly lower, while IL-18 was significantly higher in cases when COVID-19 became more severe, along with significantly decreased FVIII, FXIII and plasminogen. In the COVID-19+ group, postpartum hemorrhage (PPH) developed in 4 cases, associated with significantly reduced plasminogen, α2-plasmin inhibitor, and increased IL-8, IL-17A, IL-23 levels.

**Conclusion:**

In third trimester mild/asymptomatic COVID-19+ pregnancies, marked inflammatory cytokine changes, hemostasis alterations and enhanced fibrinolysis were found. A potential link between inflammation and PPH in the context of COVID-19 warrants further research.

## Introduction

The outbreak of the novel coronavirus (COVID-19) resulted in a global health crisis, impacting individuals across all demographics (1). Today it is well known that the disease caused by SARS-CoV-2 significantly influences the balance of hemostasis leading to a distinct entity described as COVID-19-associated coagulopathy (CAC) (2, 3). CAC involves complex interactions between the innate immune response, the coagulation and fibrinolytic pathways, and the vascular endothelium, resulting in a procoagulant condition, although the precise pathomechanism of CAC has not been fully elucidated to date (2, 4, 5). In most cases, the prothrombotic hemostasis balance present during or after the infection is accompanied by distinct laboratory features including elevated D-dimer, C-reactive protein, and fibrinogen levels as hallmarks of the disease (5). Compared with other common respiratory viral infections, patients with COVID-19 have a higher frequency and severity of thromboembolic events, moreover, accumulating evidence has shown that the risk of thromboembolism remains high for several months after the infection (6–8).

Despite the currently promising evolution of the COVID-19 pandemic, the virus continues to circulate and awareness is still suggested in vulnerable groups, including immunocompromised and pregnant individuals (9). Previous findings revealed that COVID-19 during pregnancy may be associated with an increased risk of maternal mortality and complications, particularly in the third trimester (10). Features of COVID-19-associated acute coagulopathy in pregnancy have been less extensively studied and only a handful of case reports are available reporting a comprehensive evaluation of the hemostasis system in SARS-CoV-2 infected pregnant women (11–13). The complexity of hemostasis during pregnancy may be a reason for the lack of extensive data, as physiological pregnancy is associated with changes in all aspects of hemostasis including an increase in the level of most clotting factors, decreasing concentrations of some of the natural anticoagulants and diminishing fibrinolytic activity (14, 15). At the early stages of the pandemic, international recommendations from the ISTH SSC Subcommittee on Women’s Health Issues in Thrombosis and Haemostasis provided useful consensus guidance on the management of COVID-19 coagulopathy in pregnancy, and an international registry was established to gather data to support the management of COVID-19-affected pregnant women and CAC in pregnancy (16, 17). Based on these reports and available case studies, the prothrombotic risk of COVID-19 in pregnant women has been highlighted, at the same time, awareness was raised towards increased risk of postpartum hemorrhage (PPH) (15, 18, 19). As of today, despite its potential significance, our understanding of specific hemostasis alterations in pregnant women with acute COVID-19 infection and post-COVID-19 remains limited, particularly in relation to their associations with the clinical course of pregnancy and obstetric complications, such as postpartum hemorrhage (15).

Therefore, the objective of this study was to provide a comprehensive assessment of COVID-19-associated coagulation, fibrinolysis and inflammatory cytokine/chemokine alterations in third trimester pregnancies as compared to healthy age-and gestational age-matched pregnant women. We hypothesized that pregnancies complicated by mild or asymptomatic COVID-19 exhibit distinct hemostatic and inflammatory profiles compared to healthy pregnancies. Additionally, we sought to determine potential associations between the investigated levels of hemostasis and cytokine/chemokine parameters with the clinical course of pregnancy, labor, and postpartum events.

## Methods

### Patients

In this single-center (Department of Obstetrics and Gynecology, Faculty of Medicine, University of Debrecen, Hungary) prospective, observational case-control study, pregnant women between 28-40 weeks of gestation with confirmed diagnosis of acute COVID-19 (COVID-19+ group), or with proven recovery of COVID-19 disease (post-COVID-19 group) and healthy, age- and gestational week-matched, SARS-CoV-2 negative control pregnant women were enrolled. All women were outpatients at the time of enrollment. Acute infection was confirmed/ruled out in every participant using SARS-CoV-2 RT-PCR and/or anti-SARS-CoV-2 rapid antigen test from nasopharyngeal swab samples (Genedia, St. Ingbert, Germany). Post-COVID-19 status was defined when the following criteria applied: negative SARS-CoV-2 RT-PCR and/or anti-SARS-CoV-2 rapid antigen test at least 10 days but no later than 90 days after the initial, documented SARS-CoV-2 infection (confirmed with positive SARS-CoV-2 RT-PCR and/or anti-SARS-CoV-2 rapid antigen test) (20). Patient enrollment was initiated in March 2021 and was completed in December 2022, during the 3rd, 4th and 5th wave of COVID-19, mainly dominated by the SARS-CoV-2 Delta (B.1.617.2) variant. Exclusion criteria included arterial or venous thrombotic events during pregnancy, known major thrombophilia or hemorrhagic diathesis, history of malignancy, and lack of consent.

In the COVID-19+ group, disease severity was assessed at admission according to the National Institutes of Health and World Health Organization COVID-19 clinical management guidance (asymptomatic, mild, moderate, severe or critical illness) (21).

All pregnancies were followed and detailed clinical parameters of the course of pregnancy, labor and post-partum period (including pregnancy-related complications e.g. pre-eclampsia, HELLP syndrome, delivery options: unassisted/assisted or C-section, post-partum hemorrhagic or thrombotic complications, medications, etc.) were registered up to the period of 6 weeks after childbirth. Postpartum hemorrhage was defined as a cumulative blood loss of greater than or equal to 1000 mL or blood loss accompanied by signs or symptoms of hypovolemia within 24 hours after giving birth (22). Neonatal adaptation including APGAR status of newborns was also registered.

### Informed consent

Written informed consent was obtained from all pregnant women participating in the study. Approval was obtained by the Institutional Ethics Committee of the University of Debrecen and the Ethics Committee of the National Medical Research Council (IV/3267-3/2021/EKU).

### Blood sampling and routine laboratory tests

Venous blood was drawn from all pregnant women at admission to vacutainer tubes containing ethylene diamine tetra-acetic acid (K_3_ EDTA, 3.2%, 0.105 M), sodium citrate, or serum separator (Becton Dickinson, San Jose, CA). All blood samples were drawn before the initiation of any potential therapy for the management of COVID-19 or COVID-19-related complications (including low molecular weight heparin, LMWH). All samples were processed within one hour after blood drawing. Routine laboratory tests (complete blood count, electrolytes, liver and renal function tests, total protein, albumin, C-reactive protein measurement) were carried out by standard laboratory methods. Screening tests of coagulation (prothrombin time, activated partial thromboplastin time, and thrombin time) and fibrinogen according to the method of Clauss were performed on a BCS coagulometer immediately after sample processing using standard methods (Siemens Healthcare Diagnostic Products, Marburg, Germany). For specific hemostasis assays, plasma was separated from citrated whole blood by double centrifugation (1500*g*, 15 min, room temperature). Plasma and serum samples were aliquoted and stored at -80°C until further analysis. All measurements were completed within one year after sample storage and were performed by investigators blinded to patient identification and clinical data.

### Comprehensive assessment of coagulation and fibrinolysis

Quantitative D-dimer levels were measured using a particle-enhanced, immuno-turbidimetric assay on a BCS coagulometer (Siemens Healthcare Diagnostic Products, Marburg, Germany). Factor VIII (FVIII) activity and von Willebrand factor (VWF) antigen levels were determined by standard methods as described previously (23). Plasma levels of FXIII activity were determined by ammonia release assay using a commercially available reagent kit (Technochrome FXIII, Technochlone, Austria). A sandwich ELISA assay was used to determine the levels of plasma factor XIII (FXIII-A_2_B_2_) antigen levels (24) as well as total FXIII-B subunits (25). Clot lysis assay (CLA) was performed as previously described (26). Plasminogen activator inhibitor-1 (PAI-1) activity and antigen levels were measured using Technozym PAI-1 Actibind and Technozym PAI-1 Antigen ELISA assays (Technoclone, Vienna, Austria) (27). α2-plasmin inhibitor (α2-PI) activity and plasminogen activity were determined by commercially available methods (Siemens Healthcare Diagnostic Products, Marburg, Germany).

### Thrombin generation assay

TGA was carried out using the Thrombinoscope CAT assay (Calibrated Automated Thrombogram, Maastricht, The Netherlands) according to the manufacturer’s instructions (28). Briefly, 20 μL of PPP-Reagent™ (5 pM rTF and 4 µM phospholipids, Thrombinoscope BV, Maastricht, The Netherlands) or calibrator containing thrombin-α2-macroglobulin complex (Thrombinoscope BV, Maastricht, The Netherlands) was incubated with 80 µL of plasma for 10 min in black round-bottomed 96-well microtiter plates (Greiner Bio, One North America Inc., Monroe, MI, USA). The measurement was started by automatic dispensation of 20 µL FluCa-Kit™ (a mixture of Fluorogenic substrate and Fluo-Buffer containing CaCl_2_) into each well. Fluorescence was detected by a Fluoroskan Ascent^®^ fluorimeter (Thermo Fischer Scientific, Waltham, MA) and the generated curves were analyzed by the Thrombinoscope software (Thrombinoscope BV, Maastricht, The Netherlands). The following TGA parameters were computed using the Thrombinoscope software (Thrombinoscope BV, Maastricht, The Netherlands); lag time, endogenous thrombin potential (ETP), peak thrombin, time to peak. All measurements were performed in duplicate.

### *In vitro* clot lysis assay

CLA was performed as previously described (26). Briefly, plasma samples were thawed in a water bath at 37°C. In the wells of a 96-well microtiter plate (Greiner Bio-One International GmbH, Kremsmünster, Austria), a clot induction and lysis mix was prepared in HEPES buffer (10 mM HEPES, 150 mM NaCl, 0,05% Tween20, pH 7.4), where citrated plasma was mixed with 1000-fold diluted human TF (Innovin, Siemens, Marburg, Germany) and 100 ng/mL rt-PA (Alteplase, Boehringer Ingelheim, Ingelheim, Germany). The plasma dilution with buffer was 1.2 fold. Clotting and subsequent lysis were induced by the automatic pipetting HEPES buffer containing CaCl_2_ (21 mM) into each well. Turbidity was monitored at 340 nm every minute for 300 min at 37°C using a TECAN Infinite m200 microplate reader (TECAN Trading AG, Mannedorf, Switzerland). The Shiny App software tool was used to evaluate curves (29). Clot formation and lysis were assessed using the following variables calculated from the turbidimetric curves: maximum absorbance, time to maximum absorbance, various clot lysis time (CLT) points: 10%CLT, 50%CLT, 90%CLT, and area under the curve (CLA AUC). Clot lysis times were defined as the time from the 10%, 50%, or 90% point, from clear to maximum turbidity, to the 10%, 50%, or 90% point in the transition from maximum turbidity to the final baseline turbidity, respectively. All measurements were performed in quadruplicate.

### Analysis of inflammatory cytokines

Cytokine profiling was carried out using a bead-based multiplex fluorescent immunoassay (LEGENDplex™ Human Inflammation Panel, BioLegend, San Diego, CA) according to the manufacturer’s instructions as previously described (11). The panel allowed simultaneous quantification of 13 human inflammatory cytokines (IL1-β, IFN-α2, IFN-γ, TNF- α, MCP-1, IL-6, IL-8, IL-10, IL-12p70, IL-17A, IL-18, IL-23, IL-33). The samples were read using BD FACS Canto II flow cytometer (BD Biosciences, San Jose, CA, USA), and the data were analyzed using LEGENDplex™ Data Analysis Software V8.0 (BioLegend).

### Statistical methods

Statistical analysis was performed by Graphpad Prism 9.0 software (Gaphpad Prism Inc., La Jolla, CA). Normality of data was studied using the Shapiro-Wilk-test. Continuous variables were expressed as mean ± SD or median and interquartile range (IQR). Student’s t test or Mann-Whitney U-test was performed for independent two-group analysis. Ordinary one-way ANOVA, followed by Tukey’s *post hoc* test or Kruskal-Wallis with Dunn’s *post hoc*-test were used to compare differences between groups with Gaussian and non-Gaussian distributions, respectively. Pearson’s or Spearman’s correlation coefficient was used to determine the strength of correlation between continuous variables. For all calculations, a *p*-value <0.05 was considered statistically significant.

## Results

### Baseline characteristics of the study population and results of follow-up

A total of 200 pregnant women were enrolled in the study: 100 COVID-19+ pregnant women, 32 post-COVID and 68 COVID-19-, age- and gestational-matched control pregnant women. Baseline clinical and laboratory characteristics of the cohort are shown in **Table 1**. Mean age of COVID-19+ pregnant women was 29±5 years, median gestational age was 38 (IQR: 35-39) weeks. No difference was observed between the studied groups in terms of gestational diabetes or hypertension. Based on the National Institutes of Health COVID-19 clinical management guidance, the majority of COVID-19+ pregnant women were asymptomatic or had mild illness, and only 9% had moderate or severe illness. None of the enrolled women had critical illness at admission requiring ICU care. Vaccination against SARS-CoV-2 was significantly less frequent in the COVID-19+ group (15%) as compared to the post-COVID (40%) and control (31%) groups (p=0.003). In the majority of cases, vaccination took place during the given pregnancy and not before (7/15, 8/13 and 20/20 in the COVID-19+, post-COVID-19 and control groups, respectively, with anti-SARS-CoV-2 mRNA vaccines, Pfizer-BioNTech BNT162b2 or Moderna mRNA-1273). Subtle but significant differences were observed in blood count parameters (total white blood cell count, neutrophils, eosinophils, lymphocytes, red blood cell count, hemoglobin) at admission among the groups, while platelet count was essentially similar. Among the investigated chemistry parameters only negligible differences were found between groups.

**Table 1:** Baseline clinical and laboratory characteristics of the cohort.

	COVID-19+	Post-COVID-19	Control	P value
**Number of cases**	100	32	68	–
**Maternal age, mean (**± **SD)**	29 ± 5	30 ± 5	29 ± 5	0.415
**BMI, kg/m^2^, median (IQR)**	28.2 (24.7-32.9)	29.1 (27.0-31.3)	28.7 (26.0-32.8)	0.866
Obstetrical data				
Gestational weeks, median (IQR)	38 (35-39)	39 (36-39)	38 (38-39)	0.501
Gestational diabetes, n (%)	8 (8)	4 (12)	7 (10)	0.724
Pregnancy induced hypertension, n (%)	7 (7)	2 (6)	5 (7)	0.979
Smoking, n (%)	18 (18)	1 (3)	11 (16)	0.115
Severity of COVID-19 disease*, n, (%)				
Asymptomatic	33 (33)	–	–	–
Mild illness	61 (61)	–	–	–
Moderate illness	7 (7)	–	–	–
Severe illness	2 (2)	–	–	–
Critical illness	0 (0)	–	–	–
**Vaccination against SARS-CoV-2, n (%)**	15 (15)	13 (40)	20 (29)	0.003 0.002^a^
**Days elapsed since positive SARS-CoV-2 test result, median (IQR)**	3 (1-6)	–	–	–
**Days elapsed since negative SARS-CoV-2 test result, median (IQR)**	–	25 (5-73)	–	–
Medications prior admission, n (%)				
LMWH	16 (16)	5 (16)	9 (13)	0.881
Antiplatelet	2 (2)	0 (0)	1 (1)	0.720
Antibiotics	7 (7)	1 (3)	2 (3)	0.431
Blood count parameters, median (IQR)				
WBC, G/L (total)	8.5 (6.6-10.4)	10.8 (7.8-12.0)	9.0 (7.9-10.5)	0.007 0.006^b^
Neutrophils	6.4 (4.9-7.9)	7.8 (5.8-9.1)	6.7 (5.9-8.2)	0.011 0.012^b^
Eosinophils	0.05 (0.02-0.10)	0.07 (0.05-0.11)	0.07 (0.04-0.13)	0.004 0.006^a^
Lymphocytes	1.3 (0.94-1.85)	1.7 (1.5-1.7)	1.7 (1.4-2.1)	<0.001 <0.001^a^ 0.002^b^
Monocytes	0.47 (0.33-0.63)	0.52 (0.43-0.69)	0.48 (0.43-0.55)	0.180
RBC, T/L	4.3 (3.9-4.4)	4.1 (3.7-4.4)	4.2 (4.0-4.4)	0.048 0.043^a^
Hemoglobin, g/L	120 (110-129)	124 (118-131)	127 (116-133)	0.019 0.030^a^
Platelet count, G/L	216 (172-262)	239 (193-298)	211 (170-288)	0.303
Chemistry parameters, median (IQR)				
AST, U/L	18 (15-26)	16 (14-19)	18 (16-22)	0.115
LDH, U/L	191 (173-225)	197 (167-230)	198 (183-217)	0.779
ALT, U/L	14 (10-20)	12 (8-17)	12 (9-16)	0.255
γGT, U/L	12 (9-18)	11 (7-16)	10 (7-14)	0.0240.020^a^
Serum glucose, mmol/L	4.6 (4.1-5.4)	4.6 (4.1-5.5)	4.5 (4.1-5.1)	0.747
hsCRP, mg/L	6.3 (3.4-16.7)	4.4 (3.0-12.6)	5.0 (2.7-7.8)	0.076

ALT, alanine aminotransferase; AST, aspartate aminotransferase; BMI, body mass index; γGT, gamma-glutamyl transferase; hsCRP, high-sensitivity C-reactive protein measurement; ICU, intensive care unit; IQR, interquartile range; LDH, lactate dehydrogenase; LMWH, low molecular weight heparin; n, number; RBC, red blood cell count; SD, standard deviation; WBC, white blood cell count.

*Disease severity was assessed at admission according to the National Institutes of Health COVID-19 clinical management guidance (21).

^a^COVID-19+ vs. control, ^b^COVID-19+ vs. post-COVID-19.

Results of the clinical follow-up are summarized in **Table 2**. All COVID-19+ patients recovered fully during the follow-up. After admission, COVID-19 improved or symptoms diminished in the majority of cases (95/100, 95%). COVID-19 worsened and became critical in case of five patients, who went into labor and were transferred to specific COVID-19 units within 24 hours of delivery, but fully recovered during follow-up. Median time elapsed between admission and delivery was 7 (IQR:1-15) days in the COVID-19+ group, that did not differ significantly from the time observed in the other two groups. Sixty-eight (68%) of enrolled COVID-19+ patients had active COVID-19+ disease at the time of delivery. Notably, the mode of delivery was almost identical among the groups. The frequency of obstetrical complications was low and it was similar among the investigated groups. One case of HELLP syndrome occurred in the COVID-19 group. Postpartum bleeding and preterm birth occurred in a similar rate in the COVID-19+ and in the control group. No thrombotic events occurred during pregnancy or follow-up in the investigated cohort. Prophylactic LMWH was administered during pregnancy or postpartum in 90/100 cases (90%) of COVID-19+ pregnancies as compared to 16/32 (50%) of post-COVID-19 cases and 19/68 (28%) of controls (p<0.001). Neonatal adaptation of newborns was unaltered in the COVID-19+ or post-COVID-19 group as compared to controls.

**Table 2:** Results of clinical follow up.

	COVID-19+	Post-COVID-19	Control	P value
**Number of cases**	100	32	68	–
**Time elapsed between admission and delivery, days, median (IQR)**	7 (1-15)	5 (3-15)	9 (2-17)	0.239
Mode of delivery, n (%)				
Vaginal*	74 (74)	25 (78)	50 (74)	0.875
Cesarean section	26 (26)	7 (22)	18 (26)	0.902
Obstetrical complications, n (%)				
HELLP syndrome	1 (1)	0 (0)	0 (0)	0.623
Postpartum bleeding **	4 (4)	0 (0)	3 (4)	0.356
Postpartum thrombotic event	0 (0)	0 (0)	0 (0)	–
Preterm birth	6 (6)	0 (0)	4 (6)	0.367
Neonatal adaptation, median (IQR)				
Birth weight, g	3300 (3058-3700)	3460 (3323-3948)	3420 (3195-3815)	0.118
1 min Apgar score	9 (7-10)	9 (4-10)	9 (4-10)	0.699
5 min Apgar score	10 (10-10)	10 (10-10)	10 (10-10)	0.253

HELLP syndrome, Hemolysis, Elevated Liver enzymes and Low Platelets syndrome; IQR, interquartile range; n, number.

*including assisted vaginal delivery, ** defined according to reference (22).

### Hemostatic parameters of the study cohort

#### Coagulation profile

**Figure 1:**
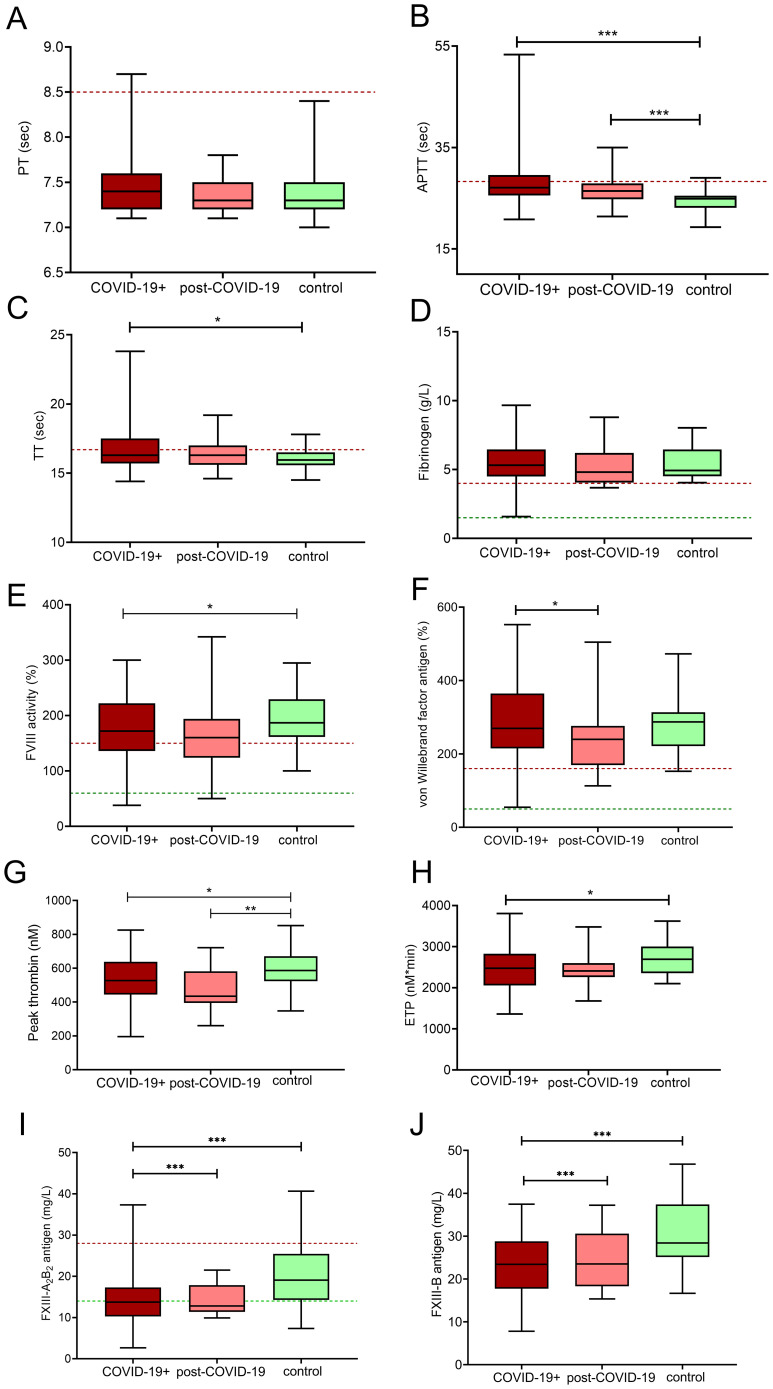
Coagulation profile in third trimester pregnant women with acute COVID-19 disease (COVID-19+, maroon boxes), post-COVID-19 (pink boxes) and healthy, age- and gestational age-matched control pregnant women (green boxes). The lower and upper box boundaries represent the 25^th^ and 75^th^ percentiles, respectively, horizontal solid lines represent the median, and whiskers indicate range. Horizontal dashed lines indicate reference ranges or thresholds determined in healthy non-pregnant individuals. Results of the screening tests of coagulation **(A–C)**, fibrinogen **(D)**, factor VIII activity **(E)** and von Willebrand factor antigen **(F)** levels, thrombin generation measurements **(G, H)**, FXIII-A_2_B_2_ antigen **(I)** and FXIII-B antigen levels **(J)** are shown. APTT, activated partial thromboplastin time; ETP, endogenous thrombin potential; FXIII A_2_B_2_, plasma factor XIII antigen; FXIII-B, factor XIII-B; PT, prothrombin time; TT, thrombin time. *p<0.05, **p<0.01, ***p<0.001. ANOVA followed by Tukey’s *post hoc* test or Kruskal Wallis with Dunn- Bonnferoni *post hoc* test.

Coagulation screening tests performed in the cohort revealed that while PT was not significantly altered in COVID-19+ pregnant women (**Figure 1A**), a significant prolongation of APTT was observed in COVID-19+ and in post-COVID-19 pregnant women as compared to healthy controls (**Figure 1B**). Significant prolongation of TT was found in COVID-19+ women as compared to healthy controls (**Figure 1C**). The median of fibrinogen levels did not differ between groups, although it must be noted that in a few patients with acute COVID-19, fibrinogen was markedly low (i.e. 1.5 g/L) (**Figure 1D**), that has probably contributed to the observed prolongation of TT as compared to healthy controls. The significant prolongation of APTT in COVID-19+ patients may be, at least partially explained by the significantly lower FVIII levels observed in this group (**Figure 1E**). On the other hand, VWF levels were significantly higher in the COVID-19+ vs. post-COVID-19 group, with considerable variation in VWF levels in COVID-19+ women (**Figure 1F**). Peak thrombin and ETP were significantly lower in COVID-19+ pregnancies as compared to healthy women (**Figures 1G, H**). FXIII-A_2_B_2_ levels and FXIII-B subunit were markedly decreased in the COVID-19+ group and in the post-COVID-19 group as compared to healthy controls (**Figures 1I, J**). Notably, in about half of COVID-19+ pregnant women FXIII-A_2_B_2_ levels were below the lower limit of reference (**Figure 1I**). The decrease in FXIII activity was parallel to the decrease in FXIII-A_2_B_2_ levels in the COVID-19+ group and the post-COVID-19 group (data not shown).

#### Fibrinolytic profile

**Figure 2:**
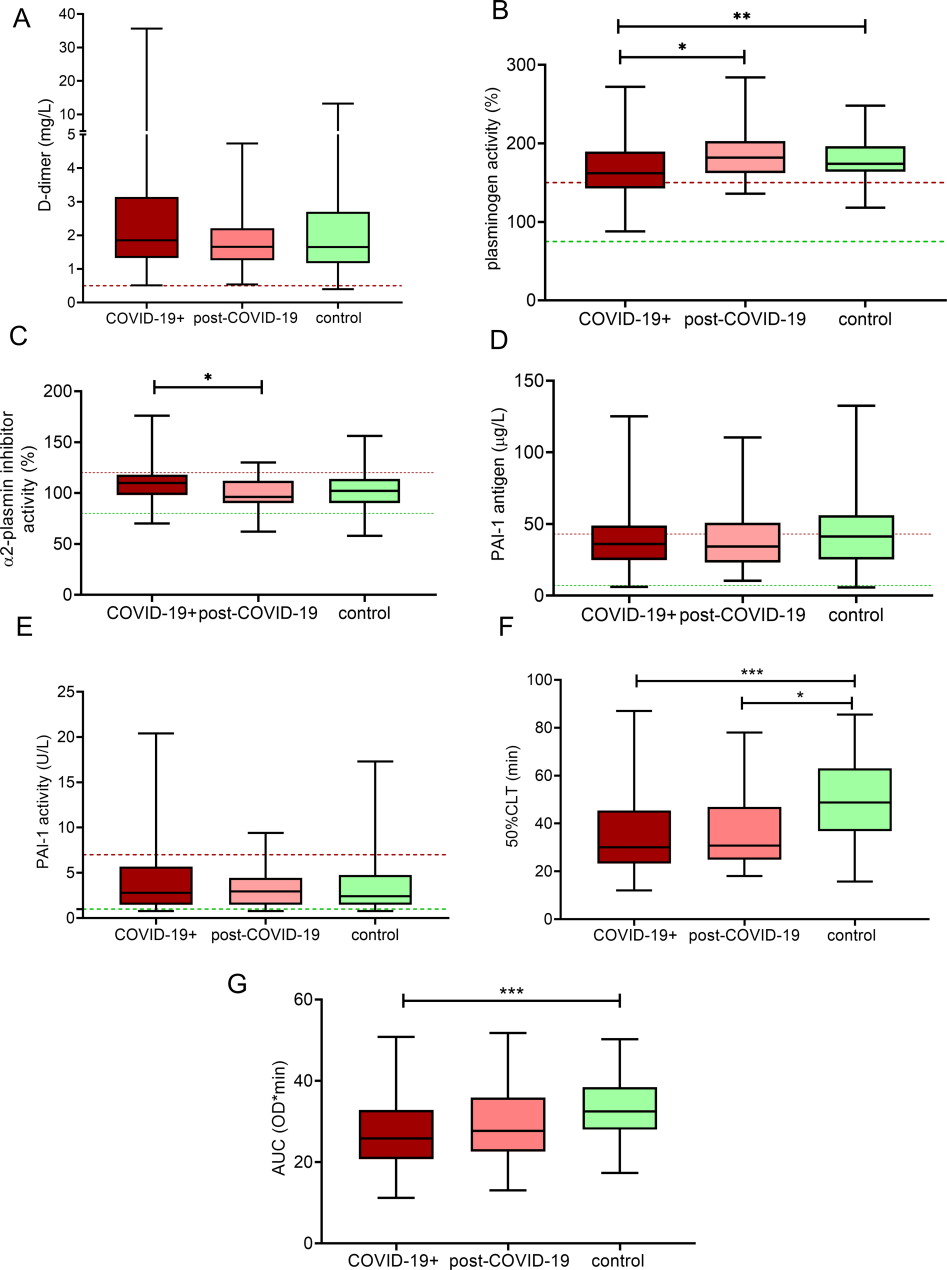
Markers of fibrinolysis in third trimester pregnant women with acute COVID-19 disease (COVID-19+, maroon boxes), post-COVID-19 (pink boxes) and healthy, age- and gestational age-matched control pregnant women (green boxes). The lower and upper box boundaries represent the 25^th^ and 75^th^ percentiles, respectively, horizontal solid lines represent the median, and whiskers indicate range. Horizontal dashed lines indicate reference ranges or thresholds determined in healthy non-pregnant individuals. Results of D-dimer **(A)**, plasminogen activity **(B)**, α2-plasmin inhibitor activity **(C)**, PAI-1 antigen **(D)**, PAI-1 activity **(E)**, 50%CLT **(F)** and AUC of CLA **(G)** are shown. 50%CLT, 50% clot lysis time; AUC, area under the curve; CLA, clot lysis assay; PAI-1, plasminogen activator inhibitor-1; *p<0.05, **p<0.01, ***p<0.001, ANOVA followed by Tukey’s *post hoc* test or Kruskal Wallis with Dunn- Bonnferoni *post hoc* test.

Interestingly, median D-dimer levels were essentially the same in all investigated groups, although in a few COVID-19+ cases the increase in D-dimer levels was more pronounced (**Figure 2A**). On the other hand, a significant decrease in functional plasminogen levels was found in the COVID-19+ group as compared to the other investigated groups (**Figure 2B**). Major inhibitors of fibrinolysis including α2-PI, PAI-1 levels were not decreased in the COVID-19+ or post-COVID-19 groups (**Figures 2C–E**). In COVID-19+ pregnant women, clot lysis was remarkably faster as compared to healthy controls (50%CLT median: 30.8, IQR: 23.3-45.8 vs. 49.9, IQR: 36.8-62.3 min, p<0.001) (**Figures 2F, G**). Notably, shorter 50%CLT as compared to controls was observed in the post-COVID-19 group as well (median: 30.8, IQR: 24.9-46.9 vs. 49.9, IQR: 36.8-62.3 min, p<0.05) (**Figure 2F**).

### Inflammatory cytokine/chemokine profile in the study cohort

**Figure 3:**
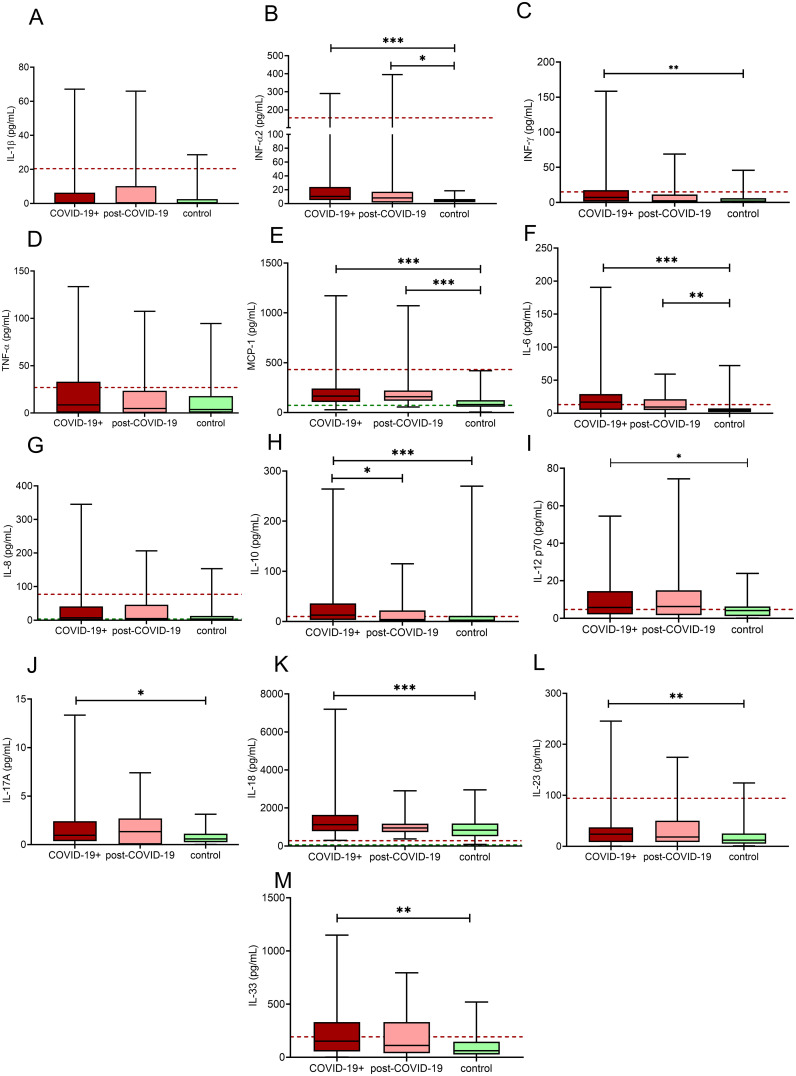
Inflammatory cytokine/chemokine in third trimester pregnant women with acute COVID-19 disease (COVID-19+, maroon boxes), post-COVID-19 (pink boxes) and healthy, age- and gestational age-matched control pregnant women (green boxes). The lower and upper box boundaries represent the 25^th^ and 75^th^ percentiles, respectively, horizontal solid lines represent the median, and whiskers indicate range. Horizontal dashed lines indicate reference ranges or thresholds determined in healthy non-pregnant individuals. Results of IL-1β **(A)**, INF-α2 **(B)**, IFN-γ **(C)**, TNF- α **(D)**, MCP-1 **(E)**, IL-6 **(F)**, IL-8 **(G)**, IL-10 **(H)**, IL-12p70 **(I)**, IL-17A **(J)**, IL-18 **(K)**, IL-23 **(L)**, IL-33 **(M)** are shown. IL, interleukin; INF, interferon, MCP-1: monocyte chemoattractant protein-1; TNF-α, tumor necrosis factor-α; *p<0.05, **p<0.01, ***p<0.001, ANOVA followed by Tukey’s *post hoc* test or Kruskal Wallis with Dunn- Bonnferoni *post hoc* test.

Comprehensive analysis of inflammatory cytokines/chemokines in the investigated cohort is demonstrated in **Figure 3**. In the COVID-19+ group, as expected, significantly increased levels of proinflammatory cytokines/chemokines including INF-α2, INF-γ, MCP-1, IL-6, IL-12p70, IL-17A, IL-18, IL-23 and IL-33 were detected (**Figures 3B, C, E, F, I–M**), while the level of IL-10, an anti-inflammatory cytokine was also elevated as compared to controls (**Figure 3H**). In the post-COVID-19 group, INF-α2, MCP-1, and IL-6 differed significantly from controls (**Figures 3B, E, F**).

### Association between inflammatory cytokine/chemokine levels and markers of coagulation and fibrinolysis in the investigated cohorts

**Figure 4:**
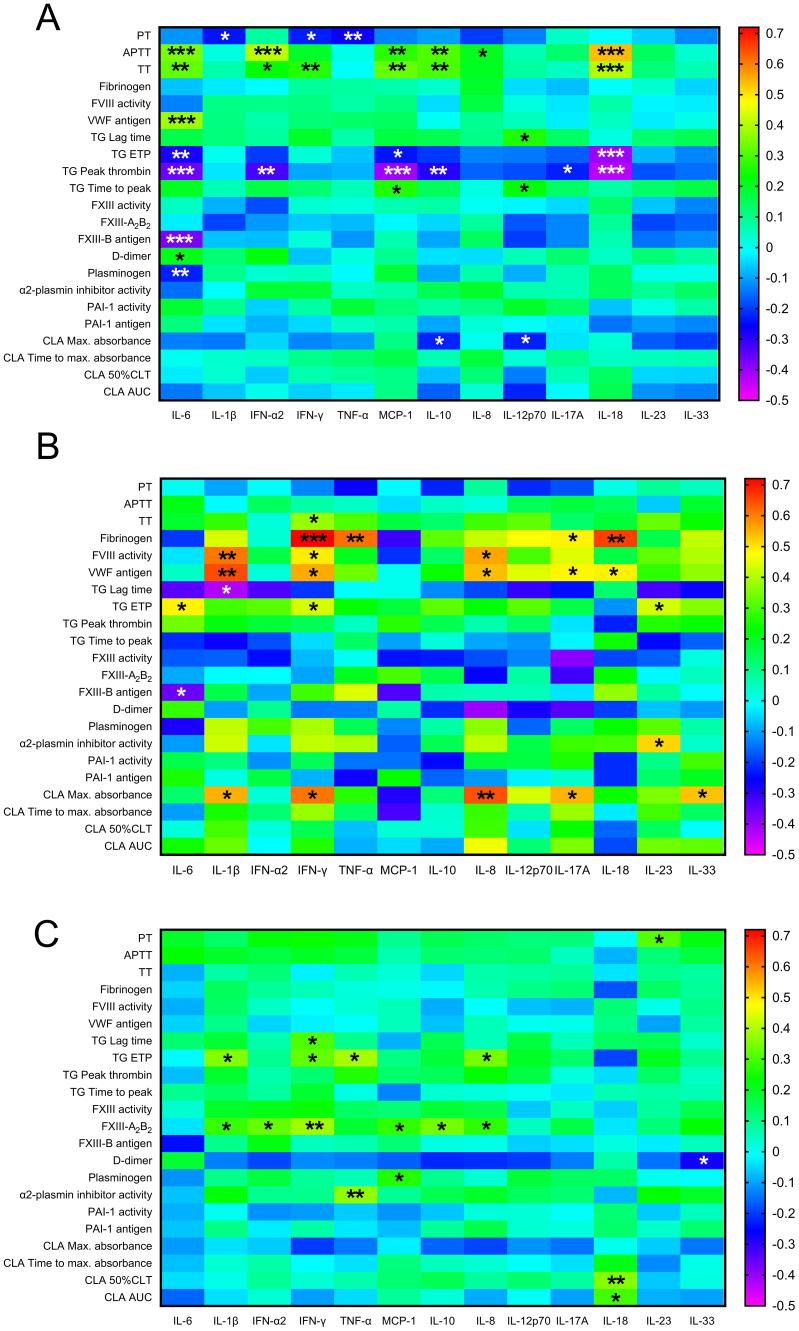
Heatmap analysis indicating correlation strength between inflammatory cytokines/chemokines and markers of hemostasis in third trimester pregnant women with acute COVID-19 disease **(A)**, post-COVID-19 **(B)** and healthy, age- and gestational age-matched control pregnant women **(C)**. Significant correlations are indicated by black/white asterisks depending on background color. *p<0.05, **p<0.01, ***p<0.001, Pearson’s or Spearman correlation.

Heatmaps derived from the correlation coefficients between all investigated hemostasis parameters vs. inflammatory cytokine/chemokine levels are shown in **Figure 4**. In pregnant women with acute COVID-19, a significant positive correlation between APTT, TT and a large subset of the tested inflammatory cytokines/chemokines was observed, including IL-6, INF-α2, MCP-1, IL-10, and IL-18 (**Figure 4A**). At the same time, significant negative association was found between the extent of TG (ETP and/or peak thrombin) and the same set of inflammatory cytokines. In contrast, VWF levels correlated positively with IL-6 only. Interestingly, only IL-6 showed significant correlation with markers of fibrinolysis in the COVID-19+ group, including a significant negative correlation with FXIII-B and with plasminogen levels and a moderate, significantly positive correlation with D-dimer. Remarkably, heatmap analysis revealed a distinct pattern of association between inflammatory cytokines and hemostasis parameters in the post-COVID-19 group (**Figure 4B**). Fibrinogen, FVIII activity, VWF levels correlated positively with a distinct subset of the tested inflammatory cytokines, including IL-1β, INF-γ, TNF-α, IL-8, and IL-18. Negative correlations between the extent of TG and inflammatory cytokines were diminished in this group, instead, moderate positive associations were revealed between ETP and IL-6, INF-γ, and IL-23. In healthy pregnant control women, only weak associations were demonstrated between the tested inflammatory cytokines and hemostasis parameters, highlighting the presence of a prominent systematic reaction in post-COVID-19, which is absent in healthy individuals (**Figure 4C**).

### Alterations of hemostasis and inflammatory markers according to COVID-19 severity

**Figure 5:**
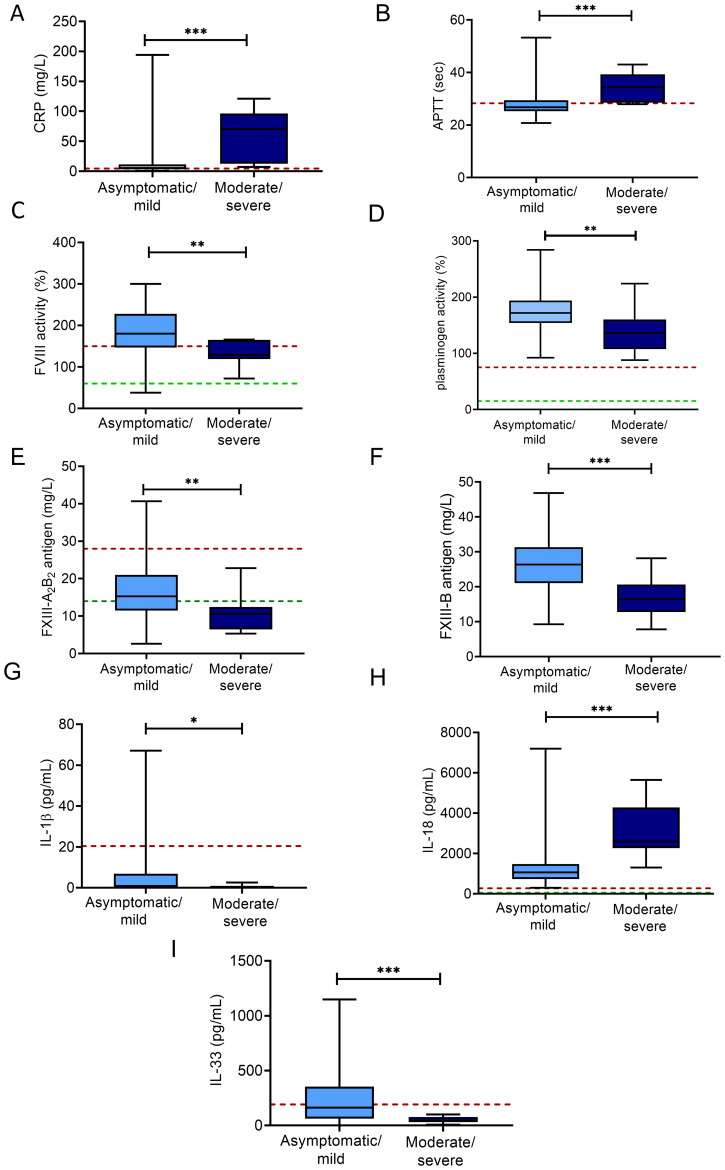
Markers of hemostasis and inflammation according to COVID-19 severity in SARS-CoV-2 infected third trimester pregnant women. Levels of CRP **(A)**, APTT **(B)**, FVIII activity **(C)**, plasminogen activity **(D)**, FXIII-A_2_B_2_ antigen **(E)**, FXIII-B antigen **(F)**, IL-1β **(G)**, IL-18 **(H)**, IL-33 **(I)** are shown in women with asymptomatic/mild COVID-19 disease (light blue boxes) vs. moderately severe/severe COVID-19 disease (dark blue boxes). The lower and upper box boundaries represent the 25^th^ and 75^th^ percentiles, respectively, horizontal solid lines represent the median, and whiskers indicate range. Horizontal dashed lines indicate reference ranges or thresholds determined in healthy non-pregnant individuals whenever available. *p<0.05, **p<0.01, ***p<0.001, Student’s t test or Mann-Whitney U test.

In patients with moderate/severe COVID-19 at admission (n=9, 9%), significantly higher levels of CRP, prolonged APTT, decreased FVIII activity, plasminogen activity, FXIII-A_2_B_2_ and FXIII-B levels were observed as compared to those with asymptomatic/mild disease (**Figures 5A–F**), while other markers of coagulation/fibrinolysis did not differ significantly between these groups. Of the tested inflammatory cytokines/chemokines, only a few significant associations were found with COVID-19 severity: lower levels of IL-1β, IL-33 together with significantly higher levels of IL-18 were found in women with moderate/severe COVID-19 as compared to asymptomatic/mild cases (**Figures 5G–I**). Interestingly, parameters demonstrating an increase in case of moderate/severe COVID-19 (e.g. CRP, APTT, IL-18) negatively correlated with the days elapsed since the positive SARS-CoV-2 test result (**Supplementary Table 1**). On the other hand, parameters demonstrating a decrease in case of moderate/severe COVID-19 (e.g. FVIII activity, plasminogen activity, FXIII and FXIII-B levels) showed a positive correlation with the days elapsed since the positive SARS-CoV-2 test result (**Supplementary Table 1**).

### Time-dependent decrease of hemostasis and inflammatory parameters in the post-COVID-19 group

**Figure 6:**
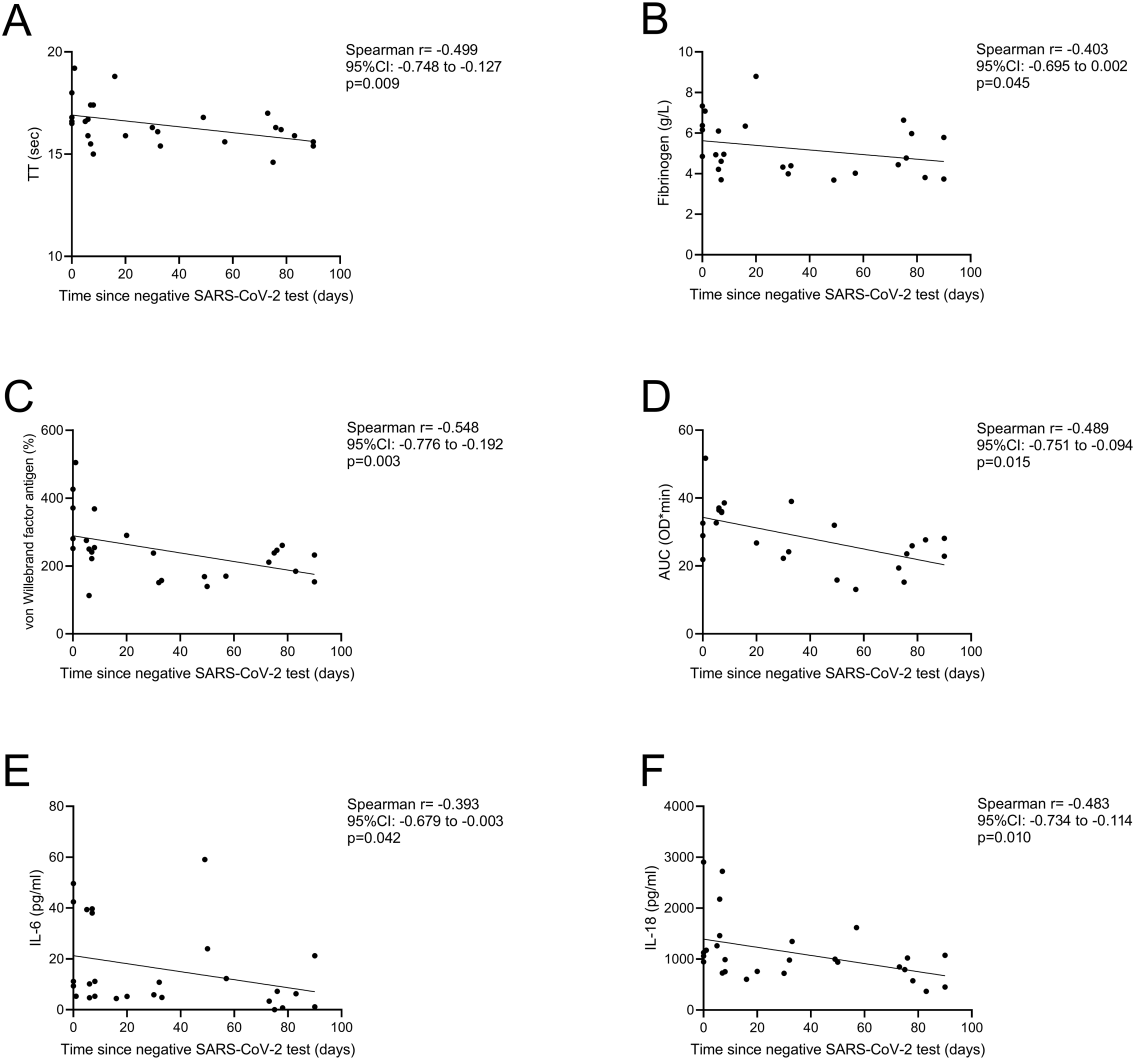
Correlation between selected hemostatic and inflammatory markers vs. days elapsed since negative SARS-CoV-2 test result in the post-COVID-19 group of pregnant women. Significant negative correlations between TT **(A)**, fibrinogen **(B)**, von Willebrand factor antigen **(C)**, clot lysis assay AUC **(D)**, IL-6 **(E)** and IL-18 **(F)** are shown. AUC, area under curve; IL, interleukin; TT, thrombin time. Spearman correlation.

**Figure 6** illustrates the levels of key hemostatic and inflammatory markers correlated with the time elapsed since a negative SARS-CoV-2 test in the post-COVID-19 group of pregnant women. Significant negative correlations between TT, fibrinogen, VWF antigen, CLA AUC, IL-6 and IL-18 vs. the time after negative SARS-CoV-2 test were observed (**Figures 6A–F**), suggesting a gradual normalization of these parameters over the period of 3 months after the negative SARS-CoV-2 test result. Other tested parameters did not yield significant correlations with the elapsed time since negative test (data not shown).

### Associations between the imbalance of hemostasis and inflammatory cytokines/chemokines and the occurrence of postpartum hemorrhage

**Figure 7:**
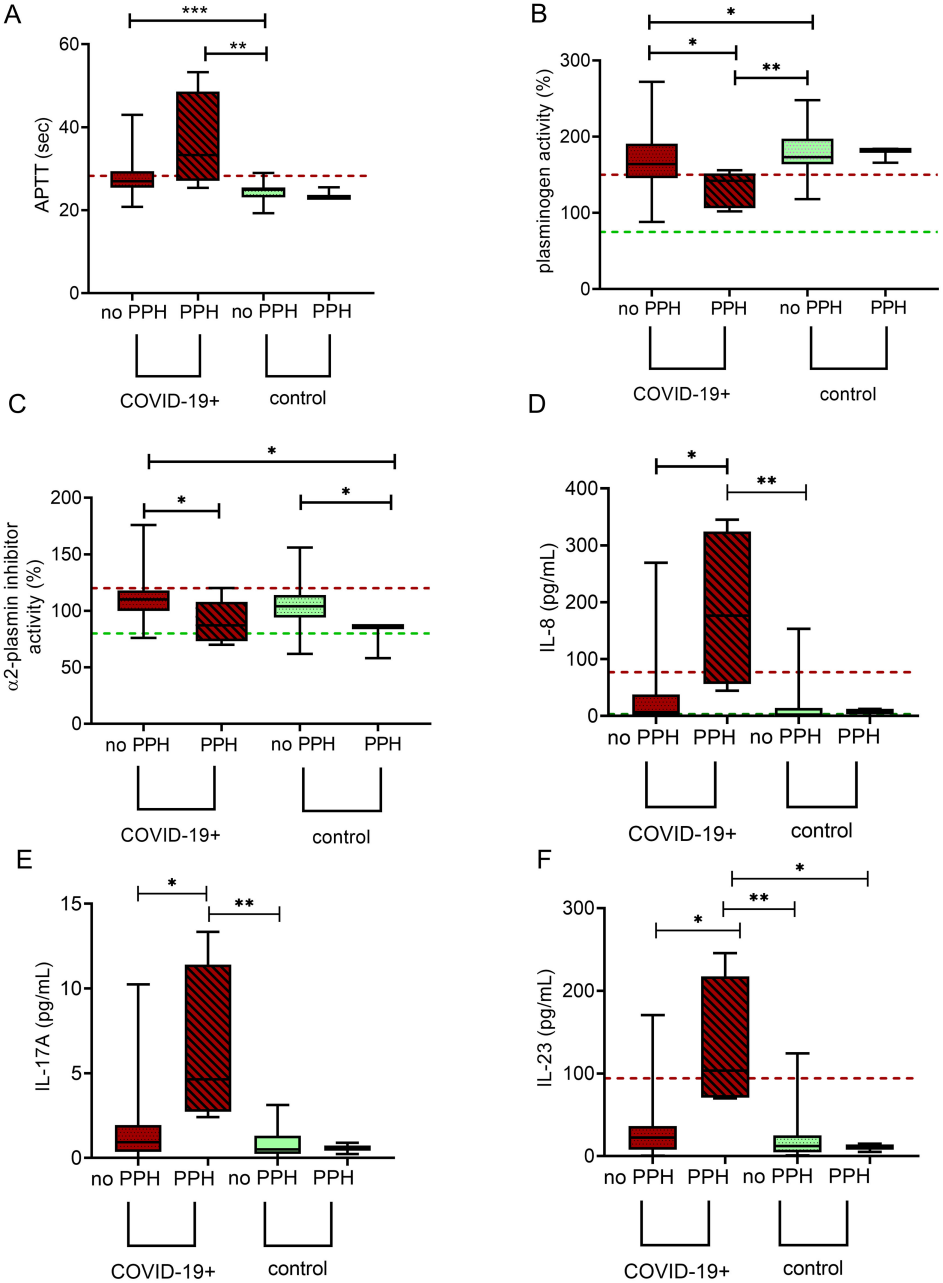
Markers of hemostasis and inflammation according to subsequent development of postpartum hemorrhage (PPH) in SARS-CoV-2 infected third trimester pregnant women as compared to healthy, age- and gestational age-matched control pregnant women. APTT **(A)**, plasminogen **(B)**, α2-plasmin inhibitor **(C)**, IL-8 **(D)**, IL-17A **(E)**, and IL-23 **(F)** levels are shown in SARS-CoV-2 infected third trimester pregnant women without (maroon boxes) or with (maroon boxes with stripe pattern) subsequent development of PPH as well as in healthy, age- and gestational age-matched control pregnant women without (green boxes) or with (green boxes with stripe pattern) subsequent development of PPH. The lower and upper box boundaries represent the 25^th^ and 75^th^ percentiles, respectively, horizontal solid lines represent the median, and whiskers indicate range. Horizontal dashed lines indicate reference ranges or thresholds determined in healthy non-pregnant individuals whenever available. APTT, activated partial thromboplastin time; IL, interleukin. *p<0.05, **p<0.01, ***p<0.001, Kruskal Wallis with Dunn- Bonnferoni *post hoc* test.

Although postpartum bleeding occurred in a similar rate in the COVID-19+ and in the control group, we hypothesized that in the COVID-19+ group, the pathophysiology of PPH might be different. All patients with COVID-19 experiencing postpartum bleeding had asymptomatic/mild COVID-19 disease. As shown in **Figure 7**, in patients with COVID-19 and PPH APTT was significantly prolonged and plasminogen levels were significantly lower as compared to women without PPH, with or without COVID-19 (**Figures 7A, B**). α2-PI was significantly lower in all women experiencing PPH, regardless of COVID-19 disease (**Figure 7C**). Other hemostasis or fibrinolytic markers, including parameters of TG or CLA did not differ significantly in this cohort of women with/without PPH. Of the tested inflammatory cytokines/chemokines, the levels of IL-8, IL-17A, and IL-23 were strikingly higher in women with COVID-19 disease and PPH as compared to those with/without COVID-19 but without PPH (**Figures 7D–F**). No difference was observed between groups in case of other inflammatory markers tested (data not shown).

## Discussion

Although the COVID-19 pandemic no longer qualifies as a global emergency, infections continue to persist in endemic forms with variable peaks (30). Thus, knowledge gathered on the pathophysiology of inflammation and hemostasis in COVID-19 may still be of value, particularly in potentially vulnerable patient cohorts, including pregnant individuals. To the best of our knowledge, our study is the first to provide detailed insights into how COVID-19 impacts hemostasis and inflammation in pregnant women and the potential association between these alterations and obstetric complications, i.e. PPH. In obstetric patients, interpretation of hemostasis alterations may be considerably more challenging due to pregnancy-induced coagulation changes, which continue to differ throughout the course of pregnancy and labor. Therefore, an essential aspect of our study was that the investigated subgroups of COVID-19+, post-COVID-19 and controls were matched in terms of age and gestational weeks. Interestingly, a series of hemostasis parameters were found to be significantly altered in COVID-19+ pregnant women as compared to healthy pregnancies, despite the fact that the majority of SARS-CoV-2 infected women in the tested cohort experienced only mild symptoms or were asymptomatic at the time of blood draw. Alterations included the prolongation APTT and TT, lower FVIII, but significantly higher VWF levels, decreased peak thrombin and ETP, markedly decreased levels of FXIII-A_2_B_2_ and FXIII-B subunit. An effect on the fibrinolytic system was demonstrated not only by the reduced FXIII levels but a notable decrease in functional plasminogen levels as well as significantly faster clot lysis as assayed by the CLA method, indicating enhanced fibrinolytic activity. In non-pregnant individuals, distinct COVID-19-related hemostasis and fibrinolysis laboratory findings, including the increase in D-dimer and VWF levels, and decrease in FXIII levels have been well-known and described in detail in the literature (31–34). Based on our study, in SARS-CoV-2 infected pregnant patients, few major differences, i.e. the lack of FVIII and D-dimer increase as compared to healthy pregnant controls can be pointed out. A potential reason for this difference may be explained by the fact that both FVIII and D-dimer increase toward term in physiological pregnancies (14), therefore, changes in their levels due to SARS-CoV-2 infection might be mitigated. On the other hand, in pregnant women with moderate or severe COVID-19 in our cohort, FVIII levels were significantly lower as compared to asymptomatic or mild carriers of the disease, a phenomenon that had not been described in non-pregnant individuals earlier. According to our findings, among patients with moderate/severe COVID-19, besides decreased FVIII activity, higher CRP levels, prolonged APTT, decreased functional plasminogen activity, and markedly reduced FXIII levels were observed compared to those with asymptomatic/mild disease. In studies on COVID-19-associated coagulopathy in non-pregnant individuals, a mechanism of consumption had been mainly accounted for the observed hemostasis and fibrinolysis alterations in COVID-19, including the cases of acquired FXIII deficiency (31–33). Although the elevation of D-dimer was not striking in COVID-19+ pregnant women in this cohort, the observed alterations are most likely a result of ongoing activation of coagulation and fibrinolysis in pregnant women as well, as a result of the complex interplay between inflammation and hemostasis.

In this study, interestingly, mild/asymptomatic COVID-19+ pregnant women exhibited significantly elevated levels of proinflammatory cytokines/chemokines such as INF-α2, INF-γ, MCP-1, IL-6, IL-12p70, IL-17A, IL-18, IL-23, and IL-33, and the anti-inflammatory cytokine IL-10. This inflammatory response was distinct from that observed in post-COVID-19 and control groups, highlighting the ongoing inflammatory process in active COVID-19. Our study revealed a complex interplay between inflammatory cytokines/chemokines and hemostatic parameters, with distinct features in acute COVID-19 and post-COVID-19. In acute COVID-19, positive correlations between APTT, TT, and several inflammatory markers (e.g., IL-6, INF-α2, MCP-1, IL-10, IL-18) were found in COVID-19+ pregnant women, at the same time, negative correlations were revealed between thrombin generation (ETP and peak thrombin) and the same set of inflammatory markers. Specific inflammatory markers (e.g., IL-6) showed significant correlations with fibrinolysis markers, highlighting the downstream connection between inflammation and fibrinolysis. Of note, heatmap analysis revealed a distinct pattern of association between inflammatory cytokines and hemostasis parameters in the post-COVID-19 group. Fibrinogen, FVIII activity, VWF levels correlated positively with a distinct subset of the tested inflammatory cytokines (e.g. IL-1β, INF-γ, TNF-α, IL-8, and IL-18), while negative correlations between the extent of TG and inflammatory cytokines were diminished in this group. Moreover, the significant negative correlations between key hemostatic and inflammatory markers and the time after negative SARS-CoV-2 test underscore the dynamic nature of COVID-19-associated inflammation and related coagulopathy, suggesting a gradual resolution of the inflammatory state induced by SARS-CoV-2 infection. These findings draw attention to an altered hemostasis balance in post-COVID-19 pregnant women, highlighting the prolonged presence of minor prothrombotic risk factors in association with inflammatory cytokines, that was not observed in healthy pregnancies.

An important question is the consequence and impact of the observed hemostasis alterations on the course of pregnancy and labor in pregnant women with COVID-19 and post-COVID-19. To date, several studies have shown that COVID-19 during pregnancy increases the risk of pregnancy complications (i.e. miscarriage, still-birth, fetal distress, premature rupture of membranes, preterm delivery, PPH) with higher risk among those with severe disease (15, 35). However, to the best of our knowledge, a potential link between hemostasis, inflammatory cytokines/chemokines and the clinical course of pregnancy in COVID-19 infected patients has not been assessed so far. The clinical follow-up in this study showed that all COVID-19+ pregnant women recovered fully. Most COVID-19+ women (95%) experienced improvement or resolution of symptoms during follow-up, however, five patients developed critical illness, necessitating labor and subsequent transfer to specialized COVID-19 units. Despite this, they fully recovered post-delivery. The median time from admission to delivery was 7 days for COVID-19+ women in this cohort, which was similar to the other investigated subgroups. The mode of delivery and frequency of obstetrical complications were comparable across all groups, indicating that COVID-19 did not significantly alter these outcomes. Notably, no thrombotic events were observed in this cohort, however, it is to be noted that prophylactic LMWH was used more frequently in COVID-19+ pregnancies compared to post-COVID-19 and control groups. Although early physician experiences indicated that CAC in pregnancy may result in thrombosis, despite prophylactic anticoagulation (16), this paradigm has been challenged today. Our observations are in line with recent studies in which the lower-than-expected incidence of thrombotic events in the SARS-CoV-2 infected pregnant population are mitigated by thromboprophylaxis and such findings underscore the importance of continuing this practice (15, 18). In this study, one case of HELLP syndrome was reported in the COVID-19 group, and the rates of postpartum bleeding and preterm birth were similar between COVID-19+ and control groups. Although the occurrence of PPH was similar between COVID-19+ and control groups, in COVID-19+ women with PPH, prolonged APTT and lower plasminogen levels were observed. Additionally, higher levels of inflammatory cytokines IL-8, IL-17A, and IL-23 were noted in these women, indicating a potential link between inflammation and PPH in the context of COVID-19, necessitating further research. According to the recent report of the ISTH registry on pregnancy and COVID-19-associated coagulopathy, PPH is one of the most frequent pregnancy complications in case of COVID-19+, affecting more than 20% of pregnant women with moderate/severe disease (18). Our results support previous findings, in which it has been suggested that an individualized approach, based on specific biomarkers, may be helpful in the future in predicting potential PPH events (36).

### Limitations

This study should be interpreted in the context of its limitations. Firstly, the study was conducted at a single center, which may limit the generalizability of the findings to other populations and settings, including different healthcare practices and pregnancy management protocols. Although the study enrolled a substantial number of pregnant participants, sample size, particularly in the post-COVID-19 group is limited, which may limit the robustness of the statistical analyses in this subgroup. The majority of pregnant women had mild or asymptomatic disease, therefore, this study may not fully capture hemostasis alterations and risks associated with moderate or severe COVID-19, limiting the applicability of findings to more severe cases. Lastly, the study included pregnant women during the pandemic waves dominated mainly by the Delta variant, therefore, the findings may not be fully applicable to other variants, including those that emerged later or may emerge in the future.

## Conclusions

This study provides comprehensive data on the impact of COVID-19 on pregnant women highlighting significant alterations in hemostatic and inflammatory profiles, despite asymptomatic or mild disease. Correlations between inflammatory markers and hemostatic parameters revealed distinct patterns in acute and post-COVID-19 groups. Detailed hemostasis results were suggestive of considerable consumption, associated with disease severity and a potential link between inflammation and PPH in the context of COVID-19. The findings of no thrombotic events in this cohort during pregnancy and follow-up underscore the importance of proactive management and prophylactic administration of LMWH in pregnant women with COVID-19.

## Data Availability

The raw data supporting the conclusions of this article will be made available by the authors, without undue reservation.
