# Supplementary material for: Complex evaluation of coagulation, fibrinolysis, and inflammatory cytokines in SARS-CoV-2 infected pregnant women: a prospective, case-control study
Source: Front Immunol. 2025 Apr 15;16:1556878. doi: 10.3389/fimmu.2025.1556878 (PMC12037393; doi:10.3389/fimmu.2025.1556878)
Supplement: Supplementary file 1 [file Table1.docx]

Supplementary Material

| **Parameters** | **r** | **95% CI** | **p value** |
| --- | --- | --- | --- |
| WBC, G/L (total) | 0.279 | 0.074 to 0.462 | 0.006 |
| Hemoglobin, g/L | 0.286 | 0.080 to 0.468 | 0.005 |
| AST, U/L | -0.234 | -0.424 to -0.024 | 0.024 |
| hsCRP, mg/L | -0.225 | -0.417 to -0.013 | 0.031 |
| PT, sec | -0.217 | -0.412 to -0.003 | 0.040 |
| APTT, sec | -0.377 | -0.548 to -0.176 | <0.001 |
| Fibrinogen, g/L | 0.212 | -0.003 to 0.409 | 0.046 |
| FVIII activity, % | 0.285 | 0.084 to 0.464 | 0.004 |
| TG Peak thrombin, nM | 0.351 | 0.143 to 0.530 | 0.001 |
| TG ETP, nM*min | 0.352 | 0.144 to 0.530 | <0.001 |
| FXIII activity, % | 0.205 | -0.004 to 0.398 | 0.048 |
| FXIII-B antigen, mg/L | 0.407 | 0.219 to 0.567 | <0.001 |
| D-dimer, mg/L | -0.203 | -0.394 to 0.003 | 0.047 |
| Plasminogen activity, % | 0.294 | 0.085 to 0.478 | 0.005 |
| CLA max. absorbance, OD | 0.283 | 0.077 to 0.466 | 0.006 |
| IL-6, pg/mL | -0.231 | -0.418 to -0.026 | 0.023 |
| IFN-**α2**, pg/mL | -0.329 | -0.502 to -0.132 | 0.001 |
| MCP-1, pg/mL | -0.362 | -0.529 to -0.168 | <0.001 |
| IL-18, pg/mL | -0.249 | -0.433 to -0.045 | 0.014 |

Supplemetary Table 1. Correlation between the days elapsed since the positive SARS-CoV-2 test result and the studied parameters in the COVID-19+ subgroup of pregnant women. Only significant correlations are shown in the Table. Abbreviations: AST, aspartate aminotransferase; APTT, activated partial thromboplastin time; CI, confidence interval; CLA max. absorbance, clot-lysis assay maximum absorbance; FVIII, factor VIII; FXIII, factor XIII; FXIII-B, factor XIII-B; hsCRP, high-sensitivity C-reactive protein measurement; IL, interleukin; IFN, interferon; MCP-1, monocyte chemoattractant protein-1; PT, prothrombin time; TG ETP, thrombin generation endogenous thrombin potential; WBC, white blood cell. Spearman correlations.
